# Prevalence of depression and its associated factors among patients with confirmed COVID-19 in Makkah, Saudi Arabia

**DOI:** 10.3389/fpsyt.2022.863215

**Published:** 2022-08-30

**Authors:** Eid Alqurashi, Ahmad Aldobyany, Abdelfattah Touman, Abdullah Alqahtani, Rajaa Alsaggaf, Omar Alnashiwaaty, Nabil Ghaleb, Hanan Mabar, Amr S. Albanna

**Affiliations:** ^1^Department of Medicine, King Abdullah Medical City, Makkah, Saudi Arabia; ^2^Department of Medicine, King Saud bin Abdulaziz University for Health Sciences, King Abdullah International Medical Research Center, Jeddah, Saudi Arabia; ^3^Department of Medicine, McGill University, Montreal, QC, Canada

**Keywords:** depression, COVID-19, SARS-CoV-2, PHQ-9, psychiatric sequalae

## Abstract

**Background:**

In early December 2019, a cluster of acute pneumonia of viral etiology had been identified in Wuhan, China. Later on, it has been named severe acute respiratory syndrome coronavirus 2 (SARS-CoV-2) causing a worldwide pandemic. This pandemic triggered unprecedented health-related psychiatric sequalae. We aim in this study to evaluate the prevalence of depression and its associated factors among confirmed patients with COVID-19.

**Methodology:**

This is a cross-sectional study, we included adult patients more than 18 years old who have been diagnosed with PCR-confirmed COVID-19 and managed in a hospital, home, or hotel. A self-administered online questionnaire based on Patient Health Questionnaire (PHQ-9) Quick Depression Assessment questionnaire was used.

**Results:**

A total of 143 subjects completed the PHQ-9 questionnaire. The prevalence of moderate to severe depression was 34%. Prevalence of depression was positively associated with the female gender (*p-*value = 0.013). Location of COVID-19 management and financial status did not affect the prevalence of depression.

**Conclusion:**

The prevalence of depression among patients with COVID-19 is high, which underscores the importance of active screening and management of depression in this population.

## Highlights

- The prevalence of depression among patients with COVID-19 is high at 33.6%.
- Prevalence of depression was positively associated with the female gender.
- Location of COVID-19 management and financial status did not affect the prevalence of depression.

## Introduction

In early December 2019, a cluster of acute pneumonia of unknown etiology had been identified in Wuhan, China. The pathogen was identified as a new RNA virus, which was named severe acute respiratory syndrome coronavirus 2 (SARS-CoV-2) (1). After the rapid global spread of this virus, the WHO declared COVID-19 a pandemic on 12 March 2020 (1).

This outbreak triggered unprecedented health-related anxiety (2). During the pandemic, the rapid spread of SARS-CoV-2 during the pandemic resulted in a great burden on health and the economy, which prompted countries and health agencies to apply strict measures to decrease viral transmission. These measures included community lockdown, social distancing, and other strict measures which resulted in psychosocial and health-related sequences.

It has been demonstrated in various research that infection outbreaks affect people’s mental and psychosocial health significantly. In the initial phase of COVID-19 spread, people started to have symptoms of anxiety especially younger individuals with chronic diseases. These psychosocial and mental symptoms increased particularly after implementing the community lockdown (3), affecting both general populations and healthcare workers. The effect was more prominent among persons who lack social support and those who have been living with a suspected case of COVID-19 (4). As opposed to an influenza outbreak, where the anxiety was ranging from 10 to 33% in the general population (5). Alsaqri and colleagues found that around 67% of the study population were suffering from a degree of social anxiety during the COVID-19 pandemic (6).

In addition to the COVID-19-related strict precautionary measures that can cause mental health problems, COVID-19 disease itself can trigger mental health disorders like depression. It has been found that the prevalence of depression was 43% in clinically stable patients with COVID-19 (7). In a systematic review that evaluated infected patients with COVID-19, the pooled prevalence of depression was 45% (8).

Different studies have shown that depression can be associated with a depressed immune system, especially cellular immunity, which may have a negative impact on COVID-19 disease progression (9). This underscores the importance of screening patients with COVID-19 for any sign of depression. Symptoms of depression can persist even after the resolution of the disease. In one study that evaluated patients with COVID-19 after one month of their discharge from the hospital, the prevalence of depression was 18% (10). A substantial percentage of patients with COVID-19 have persistent symptoms of depression 3–6 months after COVID-19 symptoms onset (11). Thus, awareness and effective management of mental health-related disorders in patients with COVID-19 is strongly required.

The location of isolation either at home or in hospital settings may also have an impact on mental health. This, however, was not sufficiently evaluated in previous studies. We aim in this study to evaluate the prevalence of depression and its associated factors among COVID-19 confirmed patients and the factors, including isolation location that may influence mental health.

## Materials and methods

This is a cross-sectional study that included adult patients more than 18 years old who have been diagnosed with SARS-CoV-2 PCR confirmed COVID-19 and managed based on Saudi ministry of health guidelines in hospitals, homes, or hotels were involved. Patients were recruited from the COVID-19 clinic over a period of 3 months (March, April, and May, 2020). Critically ill patients, patients with previous diagnoses of depression, and patients with cognitive impairment such as dementia or delirium were excluded. A self-administered online questionnaire based on Patient Health Questionnaire (PHQ-9) Quick Depression Assessment questionnaire was used. This questionnaire has been validated, and it relies on patient self-report (12, 13). An electronic questionnaire was sent to 300 patients using their e-mails and cell phones. A total of 143 patients out of 300 patients (47.7%) responded and completed the questionnaire. The questionnaire contains questions related to demographic, social, educational, and financial status. We used PHQ-9 which is categorized as the following: A score of 1–4 is minimal depression, a score of 5–9 is mild depression, a score of 10–14 is moderate depression, a score of 15–19 is moderately severe depression, and a score of 20–27 is severe depression.

## Statistical analysis

Data were analyzed by using Statistical Package for Social Studies (SPSS 22; IBM Corp., New York, NY, United States). Continuous variables were expressed as mean ± standard deviation and categorical variables were expressed as percentages. The *t*-test and the one-way ANOVA were used for continuous variables. The chi-square test was used for categorical variables. A *p*-value of <0.05 was considered statistically significant.

### Ethics and confidentiality

Ethical approval, as well as the informed consent form for the study, was taken from the KAMC Institutional Review Board (Approval number: 20-643).

## Results

A total of 143 subjects completed the PHQ9 questionnaire and were included. The baseline demographic and disease characteristics are shown in Table 1. The mean age is 41 years, and the majority (78%) of the subjects were women. Most of the subjects (73%) were diagnosed with COVID-19 for more than 1 week and half of them (49.7%) were hospitalized. Subjects with a previous diagnosis of depression represented only 2%.

**TABLE 1:** Patient’s characteristics (*n* = 143).

		Number	%
Gender	Male	65	45.5
	Female	78	54.5
Age (Mean, SD)		41.57	15.08
Education	Illiterate	16	11.2
	Primary	10	7.0
	Intermediate	14	9.8
	Secondary	29	20.3
	University	53	37.1
	Post-bachelor	21	14.7
**Chronic disease**			
DM	Yes	38	26.6
HTN	Yes	36	25.2
Heart diseases such as ischemic heart disease, heart failure, or other	Yes	6	4.2
Chronic lung disease	Yes	15	10.5
Chronic kidney disease	Yes	3	2.1
Stroke	Yes	1	0.7
Cancer (any type)	Yes	1	0.7
Other chronic diseases not mentioned above	Yes	12	8.4
When have you been diagnosed with COVID-19?	1 day	2	1.4
	2 days	3	2.1
	3 days	4	2.8
	4 days	9	6.3
	5 days	10	7.0
	6 days	10	7.0
	One week ago, or more	105	73.4
Where were you treated for COVID-19?	Stayed at home	61	42.7
	Admitted at a hospital	71	49.7
	In a hotel or other place designated for quarantine	11	7.7
What is your assessment of your current financial status?	Good	60	42.0
	Average	69	48.3
	Low	14	9.8

**FIGURE 1:**
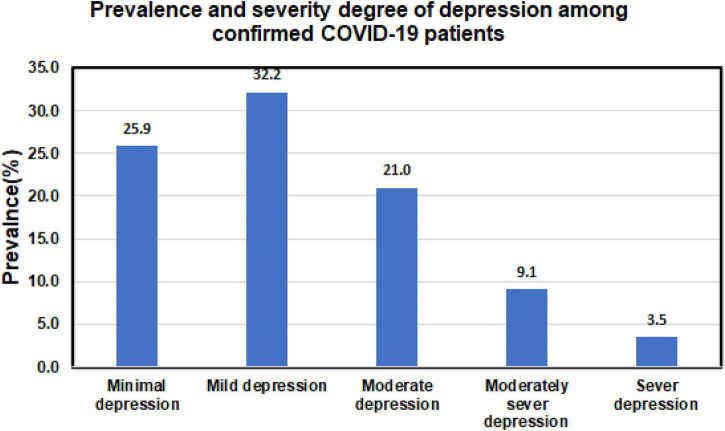
Prevalence and severity degree of depression among confirmed patients with COVID-19.

The prevalence of different depression severity categories is shown in Table 2. The prevalence of moderate to severe depression was 34%. The distribution of subjects across different degrees of depression severity is shown in Figure 1.

**TABLE 2:** The severity degree of depression among confirmed patients with COVID-19.

	Number	Prevalence (%)
Minimal depression (1–4)	37	25.9
Mild depression (5–9)	46	32.2
Moderate depression (10–14)	30	21.0
Moderately severe depression (15–19)	13	9.1
Severe depression (20–27)	5	3.5

The severity degree of depression among confirmed patients with COVID-19 by their characteristics is shown in Table 3. The mean score of PHQ-9 for confirmed patients with COVID-19 by their characteristics is shown in Table 4. The prevalence of depression was positively associated with the female gender (*p*-value = 0.013). Location of COVID-19 management and financial status did not affect the prevalence of depression.

**TABLE 3:** The prevalence and severity degree of depression among confirmed patients with COVID-19 by their characteristics.

		Minimal depression		Mild depression		Moderate depression		Moderately severe depression		Severe depression		*P*-value
		Number	%	Number	%	Number	%	Number	%	Number	%	
Gender	Male	21	32.31	17	26.15	13	20.00	4	6.15	1	1.54	0.247
	Female	16	20.51	29	37.18	17	21.79	9	11.54	4	5.13	
Education	Illiterate	5	31.25	4	25.00	1	6.25	3	18.75	1	6.25	0.137
	Primary	2	20.00	6	60.00	2	20.00	0	0.00	0	0.00	
	Intermediate	2	14.29	4	28.57	6	42.86	0	0.00	0	0.00	
	Secondary	12	41.38	4	13.79	6	20.69	2	6.90	0	0.00	
	University	9	16.98	20	37.74	11	20.75	6	11.32	4	7.55	
	Post-bachelor	7	33.33	8	38.10	4	19.05	2	9.52	0	0.00	
**Chronic disease**												
DM	Yes	8	21.05	15	39.47	4	10.53	2	5.26	2	5.26	0.274
	No	29	27.62	31	29.52	26	24.76	11	10.48	3	2.86	
HTN	Yes	8	22.22	13	36.11	6	16.67	1	2.78	2	5.56	0.484
	No	29	27.10	33	30.84	24	22.43	12	11.21	3	2.80	
Heart diseases such as ischemic heart disease, heart failure, or other	Yes		0.00	3	50.00	2	33.33	0	0.00	0	0.00	0.439
	No	37	27.01	43	31.39	28	20.44	13	9.49	5	3.65	
Chronic lung disease	Yes	3	20.00	6	40.00	2	13.33	1	6.67	0	0.00	0.801
	No	34	26.56	40	31.25	28	21.88	12	9.38	5	3.91	
Chronic kidney disease	Yes		0.00	1	33.33	2	66.67	0	0.00	0	0.00	0.425
	No	37	26.43	45	32.14	28	20.00	13	9.29	5	3.57	
Stroke	Yes		0.00	1	100.00	0	0.00	0	0.00	0	0.00	0.761
	No	37	26.06	45	31.69	30	21.13	13	9.15	5	3.52	
Cancer (any type)	Yes		0.00	1	100.00	0	0.00	0	0.00	0	0.00	0.761
	No	37	26.06	45	31.69	30	21.13	13	9.15	5	3.52	
Other chronic diseases not mentioned above	Yes	1	8.33	4	33.33	5	41.67	1	8.33	0	0.00	0.320
	No	36	27.48	42	32.06	25	19.08	12	9.16	5	3.82	
Non-chronic disease		23	28.40	22	27.16	19	23.46	9	11.11	3	3.70	0.517
When have you been diagnosed with COVID-19?	1 day	1	50.00	0	0.00	0	0.00	1	50.00	0	0.00	0.599
	2 days	0	0.00	1	33.33	1	33.33	0	0.00	0	0.00	
	3 days	1	25.00	3	75.00	0	0.00	0	0.00	0	0.00	
	4 days	2	22.22	2	22.22	1	11.11	0	0.00	1	11.11	
	5 days	3	30.00	3	30.00	2	20.00	1	10.00	0	0.00	
	6 days	1	10.00	3	30.00	1	10.00	3	30.00	0	0.00	
	One week ago or more	29	27.62	34	32.38	25	23.81	8	7.62	4	3.81	
Where were you treated for COVID-19?	Stayed at home	17	27.87	19	31.15	11	18.03	7	11.48	3	4.92	0.646
	Admitted at a hospital	15	21.13	25	35.21	16	22.54	6	8.45	2	2.82	
	In a hotel or other place designated for quarantine	5	45.45	2	18.18	3	27.27		0.00		0.00	
What is your assessment of your current financial status?	Good	17	28.33	19	31.67	11	18.33	6	10.00	2	3.33	0.979
	Average	16	23.19	24	34.78	16	23.19	6	8.70	2	2.90	
	Low	4	28.57	3	21.43	3	21.43	1	7.14	1	7.14	

**TABLE 4:** Mean score of PHQ-9 depression questionnaire for confirmed patients with COVID-19 by their characteristics.

		Mean**	SD	*P*-value
Gender	Male	6.42	4.92	0.013*
	Female	8.73	5.91	
Education	Illiterate	8.06	7.08	0.290
	Primary	7.20	3.43	
	Intermediate	7.50	5.23	
	Secondary	5.83	5.12	
	University	8.92	6.04	
	Post-bachelor	7.14	4.39	
**Chronic disease**				
DM	Yes	6.76	5.63	0.239
	No	8.01	5.55	
HTN	Yes	7.03	5.79	0.421
	No	7.90	5.52	
Heart diseases such as ischemic heart disease, heart failure, or other	Yes	7.50	5.01	0.731
	No	7.69	5.62	
Chronic lung disease	Yes	6.13	5.17	0.259
	No	7.86	5.62	
Chronic kidney disease	Yes	11.00	3.61	0.299
	No	7.61	5.60	
Stroke	Yes	6.00	0.00	0.764
	No	7.69	5.60	
Cancer (any type)	Yes	9.00		0.813
	No	7.67	5.60	
Other chronic disease not mentioned above	Yes	9.17	4.51	0.236
	No	7.54	5.66	
When have you been diagnosed with COVID-19?	1 day	9.00	9.90	0.874
	2 days	6.67	6.11	
	3 days	5.25	3.30	
	4 days	6.22	8.00	
	5 days	6.70	4.90	
	6 days	8.90	6.59	
	One week ago or more	7.88	5.39	
Where were you treated for COVID-19?	Stayed at home	7.84	6.09	0.519
	Admitted at a hospital	7.83	5.30	
	In a hotel or other place designated for quarantine	5.82	4.38	
What is your assessment for your current financial status?	Good	7.45	5.68	0.845
	Average	7.96	5.51	
	Low	7.29	5.85	
Overall score		7.68	5.58	

*Significant *p*-value.

**Out of 27.

## Discussion

We found that around one-third (34%) of patients diagnosed with COVID-19 suffered from moderate to severe depression, which was positively associated with the female gender. Location of COVID-19 management (hospital, home, or hotel) and financial status did not affect the prevalence of depression.

Our findings are consistent with what has been found in other studies. Ma and colleagues found that the prevalence of depression among patients with COVID-19 using the PHQ-9 questionnaire was 43.1%. Female gender and family history of severe COVID-19 disease have been found to be strong predictors of depression (7). In another cross-sectional study that was conducted on 1,002 patients with COVID-19 using PHQ-9, the prevalence of moderate to severe depression was as high as 48%. Depression was positively associated with lower family income, sleep disturbance, lack of physical activity, fear of COVID-19 re-infection, and persistent COVID-19 symptoms (14). In our study, female gender was a significant risk factor for depression. Other factors including financial status, comorbidities, duration of having COVID-19, and location of isolation did not affect the prevalence of depression.

Prevalence of depression has been found to be higher in patients with COVID-19 as compared to the general population. Alamri and colleagues found that the prevalence of depression among the general population in Saudi Arabia during the COVID-19 pandemic is 17% (15). It is low as compared to our finding in this study which is 34%. In a systematic review that involved multiple studies in different countries, the overall prevalence of depression in the general population was ranging from 14.6 to 48.3% (16). While in patients with COVID-19, the prevalence was higher as demonstrated in a systematic review and meta-analysis that evaluated infected patients with COVID-19, which found that the pooled prevalence of depression was 45% (8).

During the COVID-19 pandemic prevalence of depression was relatively high compared to the pre-pandemic period. The prevalence of depression symptoms in the United States of America was more than threefold higher during the COVID-19 pandemic compared to the pre-pandemic period (17). In one survey of the general population before the COVID-19 pandemic in Saudi Arabia, the prevalence of depression was 3.8% (18), which increased significantly during the pandemic as indicated in this study is 34%.

Some studies showed a higher prevalence of depression in actively hospitalized patients. Samrah and colleagues did a survey on patients with COVID-19 after 10 days of hospital isolation which showed a very high prevalence of 44% (19). In another study, 97.2% of hospitalized patients with COVID-19 with stable conditions had some degree of depression (20). In our study, the location where the patient received COVID-19 management at home, hotel, or hospital did not affect the prevalence of depression.

Although the high prevalence of depression among patients with COVID-19 could be explained by the quarantine and fear of disease, the inflammatory process of COVID-19 has been found to play a role in the psychiatric sequalae. Baseline systemic immune-inflammation index has positively associated with the prevalence of depression in 402 adults surviving COVID-19 at 1-month follow-up after hospital treatment (21).

It is important to acknowledge the limitations of our study, which include small sample size and an online self-assessment questionnaire rather than a face-to-face meeting and evaluation. Also, the absence of a control group from the general population during the same period is one of the limitations. On the other hand, we used a well-validated questionnaire and assessed different important variables that might affect the mental status.

Our study findings have important clinical implications as they indicate that the prevalence of depression is high in patients with COVID-19 as compared to the general population during the COVID-19 pandemic. This raises the importance of good assessment and evaluation of those patients for health-related psychiatric sequalae. This can be conducted through telemedicine or web-based care. In one study, digitally enabled remote care for people with long COVID-19 syndrome showed promising results; and there are ongoing studies to assess the online cognitive behavioral therapy that will be more accessible with less cost (22–24).

## Conclusion

The prevalence of depression among patients with COVID-19 is high (34%), which underscores the importance of active screening and management of depression in this population that can be provided through telemedicine or web-based care. Depression was positively associated with the female gender in our population; however, the location of COVID-19 management and financial status did not influence the prevalence of depression.

## Data availability statement

The raw data supporting the conclusions of this article will be made available by the authors, without undue reservation.

## Ethics statement

The studies involving human participants were reviewed and approved by The Institutional Review Board (IRB) of King Abdullah Medical City (KAMC) (Approval number: 20-643). The patients/participants provided their written informed consent to participate in this study.

## Author contributions

EA and AA designed and supervised the study. EA, AMA, AT, ASA, and RA contributed to protocol development, data collection, interpretation of the analyzed data, and manuscript writing. OA, NG, and HM were involved in data collection and entry as well as manuscript editing. All authors reviewed and approved the final version of the manuscript.
